# Rational design of cisplatin and carboplatin complexes for enhanced anticancer efficacy based on DFT QTAIM and docking analyses

**DOI:** 10.1038/s41598-025-34380-x

**Published:** 2026-01-07

**Authors:** Mohammed Ghazwani, Umme Hani

**Affiliations:** Department of Pharmaceutics, College of Pharmacy, King Khalid University, Al Faraa, 62223 Abha, Saudi Arabia

**Keywords:** Cisplatin, Carboplatin, Hybrid complexes, Drug synergy, DFT, Molecular docking, Biochemistry, Cancer, Chemical biology, Chemistry, Computational biology and bioinformatics, Drug discovery

## Abstract

Cisplatin (CP) and carboplatin (CBP), two key platinum‑based anticancer drugs, face clinical limitations that prompt the search for new strategies to enhance efficacy and reduce toxicity. This study applies density functional theory (DFT), quantum theory of atoms in molecules (QTAIM), molecular docking, and spectroscopic analyses to explore possible synergistic effects of cisplatin–carboplatin [CP–CBP] complexes in breast and cervical cancers. Structural optimizations show small bond‑length adjustments in the [CP–CBP] complexes, which strengthen intermolecular interactions and overall stability. Thermodynamic analyses confirm their exothermic nature (ΔH < 0), indicating thermodynamic stability, while adsorption energies (Ead = − 14.69, − 12.47, − 14.27 kcal/mol for States I, II, III) suggest enhanced bioavailability and controlled release in aqueous environments, though higher gas-phase energies indicate stronger interactions. Quantum descriptors, including electrophilicity index (ω) and chemical potential (μ), reveal increased reactivity and improved drug-target interactions, supporting enhanced anticancer potential. Spectroscopic analyses (UV–Vis, IR) confirm altered electronic transitions, reinforcing stability and reactivity changes. Molecular docking indicates that [CP–CBP] complexes outperform individual CP and CBP, with State III achieving − 3.75 kcal/mol (Ki = 1.78 μM) for aromatase and State II − 5.48 kcal/mol (Ki = 96.86 μM) for HER2. CBP stabilizes CP, preventing degradation, enhancing solubility, and enabling controlled release, reducing toxicity. These findings highlight [CP–CBP] complexes as a promising platinum‑based chemotherapeutic strategy with potentially improved pharmacokinetics, warranting further in vitro and in vivo validation for targeted cancer therapy.

## Introduction

Cancer, characterized by the uncontrolled proliferation and metastasis of abnormal cells, remains a leading cause of mortality worldwide, particularly in developing countries^1^. Platinum-based chemotherapy, a cornerstone in cancer treatment, is widely employed for malignancies such as cervical, ovarian, lung, and colon cancers^2–4^. Among these agents, cisplatin (CP), first synthesized in 1845 and FDA-approved in 1978, stands as a pioneering chemotherapeutic drug^5,6^. Despite its efficacy, cisplatin’s clinical utility is hampered by severe adverse effects, including nephrotoxicity, neurotoxicity, and myelosuppression^7–11^. To address these limitations, carboplatin (CBP), a second-generation platinum analog with reduced toxicity, was introduced in 1981^12,13^. However, carboplatin’s therapeutic profile is still challenged by peripheral nerve damage, nephrotoxicity, and a short plasma half-life, which restrict its clinical applicability^14,15^.

A promising strategy to enhance therapeutic efficacy while minimizing toxicity involves the use of drug delivery systems. Such systems can protect platinum drugs from inactivation, prolong circulation time, and facilitate targeted tissue delivery^16–19^. Encapsulation or conjugation of these drugs within carriers, through physical adsorption or chemical bonding, has shown potential to reduce hydrolysis and protein‑mediated deactivation^20,21^. Notably, encapsulating CBP within molecular carriers like Cucurbituril could prevent its conversion to toxic CP derivatives in chloride-rich biological environments, thereby improving safety^22^. Concurrently, combination therapies leveraging synergistic interactions between chemotherapeutic agents have emerged as a strategy to overcome drug resistance and amplify anticancer effects^23^. Computational approaches, such as density functional theory (DFT) and molecular docking, have proven instrumental in elucidating drug-drug interactions and optimizing delivery systems^23,24^.

However, existing research has predominantly focused on individual platinum drugs or their pairing with non-platinum agents, leaving a critical gap in understanding the combined use of CP and CBP. In current clinical practice, cisplatin and carboplatin are not used together, as both belong to the same class of platinum-based drugs and are typically employed as therapeutic alternatives rather than in combination. To date, no approved treatment protocols or clinical studies have explored their concurrent use. Therefore, this study theoretically investigates whether the formation of a hybrid cisplatin–carboplatin [CP–CBP] complex could offer a rational strategy to integrate the complementary properties of both drugs. Cisplatin provides potent cytotoxic activity but suffers from severe side effects, while carboplatin exhibits reduced toxicity yet lower potency. By forming [CP–CBP] complexes, carboplatin may act as a stabilizing, carrier‑like component that prevents premature degradation of cisplatin, enhances solubility, and enables controlled release, while cisplatin compensates for the lower anticancer potency of carboplatin. Thus, this combination is not arbitrary, but rather a rational attempt to synergistically exploit their complementary properties for improved therapeutic outcomes. This study is therefore significant as it bridges a critical gap in platinum-based chemotherapy research by proposing a hybrid [CP–CBP] complex that combines the stability of carboplatin with the potency of cisplatin. Unlike previous works that evaluated each drug separately, this work provides an integrated theoretical framework supported by DFT, QTAIM, and docking validations, aiming to rationalize synergistic effects at the molecular level.

To address this research gap, the present study aims to comprehensively evaluate the feasibility of [CP–CBP] interactions in cancer treatment, with a specific focus on breast and cervical cancers. This research investigates the physicochemical properties of the CP–CBP complexes and explores its potential therapeutic applications. By examining the role of CBP as a carrier-like for CP, the study delves into the combined therapeutic potential of these two agents through complexes formation, emphasizing their synergistic effects and advantages over monotherapy. Molecular docking studies, widely used for predicting drug interactions with protein targets, offer insights into the binding orientation and conformation of drug molecules within active sites^23^. Given the complexity and cost-intensive nature of drug development, computational docking serves as a valuable tool for predicting pharmacological activity before synthesis. Therefore, in this research, molecular docking analyses were conducted to evaluate the combined effect of the CP–CBP complexes as a potential inhibitor in the treatment of breast (PDB ID: 3RCD) and cervical (PDB ID: 3EQM) cancers. The physicochemical properties, stability, and reactivity of the complexes were assessed through various computational methods, including DFT, quantum molecular descriptors, and Quantum Theory of Atoms in Molecules (QTAIM) analysis^25–29^. Additionally, UV–Vis and infrared spectra were simulated to understand the photochemical and vibrational behavior of the complexes. These analyses aim to provide insights into the potential of CP–CBP complexes as effective chemotherapeutic agents, enhancing efficacy while reducing toxicity.

## Computational method

### Density functional theory (DFT) optimization

To comprehensively investigate the structural, electronic, and pharmacological properties of the CP–CBP complexes for their potential application in breast and cervical cancer treatment, a series of computational techniques were employed. The initial structures of CP and CBP were obtained from PubChem and optimized using DFT. Geometry optimization was performed with the BP86-D3^30^, mPW1PW91-D3^31^, and PBE1PBE-D3^32^ functionals, which include Grimme’s dispersion correction (D3BJ) to accurately account for van der Waals interactions^33^. For platinum atoms, the LANL2DZ basis set was utilized due to its suitability for transition metals^34^, while the H, N, Cl, C, and O atoms were modeled using the 6–31 + G** basis set. To ensure the reliability of the computational setup, the optimized geometries of cisplatin and carboplatin obtained using the BP86-D3/LANL2DZ/6–31 + G level were cross-checked with experimental crystallographic data reported in the literature^2,3,5^. The validation details and corresponding comparison are presented in Table 2. This benchmarking step confirmed that the selected functional and basis sets are appropriate for accurately describing Pt–N, Pt–Cl, and Pt–O coordination environments in platinum complexes. Frequency calculations confirmed the absence of imaginary frequencies, ensuring that the optimized structures corresponded to true minima on the potential energy surface. These optimizations were carried out both in the gas phase and under solvation conditions using the polarizable continuum model (PCM) with the solvation model density (SMD) approach for water^35^. The adsorption energy (Ead) of the CP–CBP complexes was calculated to evaluate their stability and interaction strength. The formula used for this calculation is as follows:^25^

1 $${\mathrm{E}}_{{{\mathrm{ad}}}} = \, \left( {{\mathrm{E}}_{{{\mathrm{CP}}/{\mathrm{CBP}}}} + {\text{ ZPE}}} \right) \, {-} \, \left( {{\mathrm{E}}_{{{\mathrm{CP}}}} + {\text{ ZPE}}} \right) \, {-} \, \left( {{\mathrm{E}}_{{{\mathrm{CBP}}}} + {\text{ ZPE}}} \right) \, + {\text{ E}}_{{{\mathrm{BSSE}}}}$$

Here, E_CP/CBP_ , E_CP_, and E_CBP_ represent the electronic energies of the complex, CP, and CBP, respectively. ZPE denotes zero-point energy, and _EBSSE_ refers to the basis set superposition error correction obtained via the counterpoise method^36^. All calculations were performed using Gaussian 09 software^37^, ensuring accurate corrections for intermolecular interactions.

### Quantum molecular descriptors

Quantum molecular descriptors (QMDs) were computed to assess the reactivity and stability of the CP–CBP complexes. These included electron affinity (A), ionization potential (I), global softness (S), chemical potential (μ), electronegativity (χ), global hardness (η), and electrophilicity index (ω). The equations used for these calculations are as follows:

2 $$\mu = - \frac{1}{2}\left( {I + A} \right)$$

3 $$\chi = - \mu$$

4 $$\eta = \frac{1}{2}\left( {I - A} \right)$$

5 $$S = \frac{1}{2\eta }$$

6 $$\omega = \frac{{\mu^{2} }}{2\eta }$$

These parameters provided insights into the electronic structure and chemical behavior of the complexes, offering a deeper understanding of their potential pharmacological activity^38^.

### Thermodynamic analysis

Thermodynamic properties such as enthalpy (ΔH), Gibbs free energy (ΔG), and entropy changes (ΔS) were calculated using frequency analysis at standard conditions (1 atm pressure, 298.14 K temperature). The equations used for these calculations are:

7 $$\Delta {\text{H }} = {\text{ H}}_{{{\mathrm{CP}}/{\mathrm{CBP}}}} {-}{\text{ H}}_{{{\mathrm{CP}}}} {-}{\text{ H}}_{{{\mathrm{CBP}}}}$$

8 $$\Delta {\text{G }} = {\text{ G}}_{{{\mathrm{CP}}/{\mathrm{CBP}}}} {-}{\text{ G}}_{{{\mathrm{CP}}}} {-}{\text{ G}}_{{{\mathrm{CBP}}}}$$

9 $$\Delta {\text{G }} = \, \Delta {\text{H }}{-}{\text{ T}}\Delta {\mathrm{S}}$$

These thermodynamic evaluations helped determine the feasibility of CP–CBP complex formation under physiological conditions^39^.

The energy gap (Eg) between the Highest Occupied Molecular Orbital (HOMO) and the Lowest Unoccupied Molecular Orbital (LUMO) was also determined to analyze the electronic characteristics of the systems:

10 $${\mathrm{E}}_{{\mathrm{g}}} = {\text{ E}}_{{{\mathrm{HOMO}}}} - {\text{ E}}_{{{\mathrm{LUMO}}}}$$

This parameter is critical for understanding the stability and reactivity of the complexes, providing valuable information about their potential interaction with biological targets.

### Quantum theory of atoms in molecules (QTAIM) and related analyses

Quantum Theory of Atoms in Molecules (QTAIM), Electron Localization Function (ELF), and Localized Orbital Locator (LOL) analyses were performed using Multiwfn software^40^ to gain further insights into the bonding patterns, electron localization, and interaction mechanisms within the CP–CBP complexes. These analyses enhanced our understanding of the structural integrity and intermolecular interactions of the complexes.

### UV–vis and infrared (IR) spectroscopy

UV–Vis spectra and infrared (IR) analysis were conducted to study the photochemical behavior and vibrational characteristics of the CP–CBP complexes. These analyses provided insights into the electronic transitions and molecular vibrations, which are critical for understanding how the complexes interact with biological environments under varying conditions. The UV–Vis spectra were simulated using Time-Dependent DFT (TD-DFT)^41^ with the BP86 functional 30], while IR spectra were calculated based on harmonic vibrational frequency analysis at the same level of theory used for geometry optimization. Both studies were performed in both gas and aqueous phases to account for environmental effects on the spectral properties.

### Molecular docking simulations

Molecular docking simulations were conducted to explore the binding modes and affinities of the CP–CBP complexes with key protein targets implicated in breast and cervical cancers. Crystal structures of human epidermal growth factor receptor 2 (HER2, PDB ID: 3RCD) and human placental aromatase cytochrome P450 19A1 (PDB ID: 3EQM) were downloaded from the Protein Data Bank. Missing residues and structural irregularities in the protein structures were repaired using GalaxyRefine^42^ to ensure geometric optimization. The active sites were identified based on prior crystallographic data and literature reports. Preprocessing involved removing water molecules, adding Gasteiger partial charges, and introducing polar hydrogen atoms using AutoDock Tools (version 4.2)^43^. Docking calculations were carried out in AutoDock 4.2 using the Lamarckian genetic algorithm. A cubic grid box (40 × 40 × 40 Å, spacing 0.375 Å) was centered on the active site of each receptor (3RCD: x = 10.194, y = 1.333, z = 29.833 Å; 3EQM: x = 87.881, y = 56.236, z = 42.031 Å), with a population size of 150 and 100 GA runs. Docking results were visualized and analyzed using Discovery Studio Visualizer^44^ to identify key interactions and evaluate binding affinities. By integrating these advanced computational techniques, this study provides a robust framework for understanding the physicochemical and pharmacological properties of the CP–CBP complexes, paving the way for their potential development as novel chemotherapeutic agents in the treatment of breast and cervical cancers.

## Results and discussion

To improve readability, this section first provides a brief overview of the main findings and then presents detailed analyses for each group of results. Specifically, Sect. “The optimized Structures of CP and CBP drugs and their complexes” discusses the optimized geometries and thermodynamic stability of CP, CBP, and their complexes; Sect. “Quantum chemical analysis of CP–CBP complexes: insights from QTAIM, ELF, and LOL” focuses on bonding and electron-density analyses (QTAIM/ELF/LOL); Sects. “UV–Vis analysis and frontier molecular orbitals” and “Infrared Spectroscopy analysis of molecular interactions and structural changes in CP–CBP complexes” describe the optical (UV–Vis) and vibrational (IR) spectra; and Sect. “Molecular docking analysis of CP, CBP, and their complexes for breast and cervical cancer treatment” reports the docking interactions with HER2 and aromatase.

### The optimized structures of CP and CBP drugs and their complexes

The optimized structures of CP and CBP were determined using three different computational methods: BP86-D3^30^, mPW1PW91-D3^31^, and PBE1PBE-D3^32^. These methods were employed to identify the most suitable functional for subsequent calculations, ensuring accurate representation of the molecular geometries and energy profiles of the drugs. The results obtained from these optimizations are summarized in Table 1, which presents the sum of electronic and zero-point energies (Hartrees) for both CP and CBP in the gas phase. Based on the results presented in Table 1 and previous studies^45^, the BP86-D3 functional was identified as the most appropriate method for describing the electronic structure of CP and CBP due to its consistent performance across both compounds. Consequently, all subsequent calculations, including geometry optimization, frequency analysis, and adsorption energy evaluations, were carried out using the BP86-D3/LANL2DZ/6–31 + G** level of theory. This approach ensures the highest degree of accuracy and reliability in predicting the physicochemical properties of the CP–CBP complexes.

**Table 1:** Sum of electronic and zero-point Energy (Hartrees) of CP and CBP drugs in the gas phase.

Properties	Method	CP	CBP
Energy + ZPE	BP86-D3/LANL2DZ/6–31 + G**	− 1152.869668	− 765.433500
	mPW1PW91-D3/LANL2DZ/6–31 + G**	− 1152.729039	− 765.193258
	pbe1pbe-D3/ LANL2DZ/6–31 + G**	− 1152.228315	− 764.587276

**Fig. 1:**
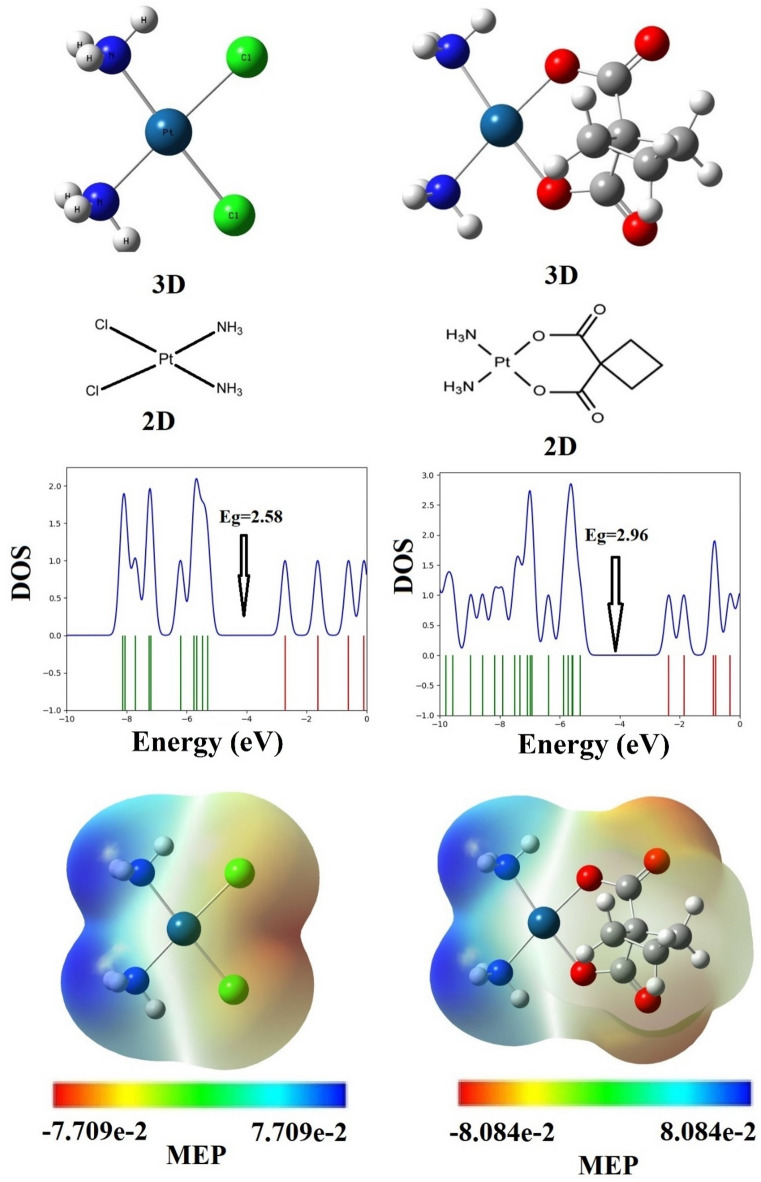
Structures optimized (3D) and 2D visual representations, DOS, and MEP plots of the CP and CBP drugs.

The optimized structures of CP and CBP in gas and aqueous phases reveal distinct adaptations, as shown in Table 2. In the gas phase, CP exhibits Pt–N distances of approximately 2.108–2.109 Å and Pt-Cl distances of 2.342–2.343 Å, which change in water to 2.079 Å and 2.379 Å, respectively. For CBP, Pt–N and Pt-O distances in gas phase are 2.109–2.110 Å and 2.014–2.015 Å, slightly reducing to 2.081 Å and 2.046 Å in solution, indicating enhanced Pt-ligand interactions due to solvation effects. Comparison with experimental crystallographic data shows reasonable agreement with computational results. The experimentally determined Pt–N and Pt-Cl distances for CP are reported as approximately 2.05–2.08 Å and 2.32–2.35 Å, respectively [2 and 3]. Similarly, for CBP, Pt–N and Pt–O distances have been reported in the range of 2.00–2.10 Å and 2.02–2.05 Å, respectively^5^. These comparisons validate the reliability of the computational approach in predicting structural parameters relevant to the physicochemical behavior of CP and CBP in different environments. Additionally, Fig. 1 presents the graphical representations of the optimized structures, including both 3D models and 2D schematic diagrams of CP and CBP. The figure also includes the density of states (DOS) plots, illustrating the electronic distribution, and molecular electrostatic potential (MEP) maps, highlighting charge distribution across the molecules. These visualizations help in understanding the electronic properties and structural adaptations of the drugs in different phases. Similar approaches have been reported in recent DFT and MEP studies, where molecular electrostatic potential mapping was used to locate nucleophilic and electrophilic regions and to support drug–target reactivity analysis^46,47^.

**Table 2:** The bond lengths, adsorption energy (E_ad_), solvation energy (E_sol_), enthalpy change (ΔH), Gibbs free energy change (ΔG), entropy change (ΔS), dipole moment (DM), energy values of HOMO and LUMO, gap energy (E_g_), and Quantum Molecular Descriptors of cisplatin (CP) and carboplatin (CBP) drugs and as well as State I, II, and III in the [CP–CBP] complexes in the gas and water environments.

Solvent	Gas	Water	Gas	Water	Gas	Water	Gas	Water	Gas	Water
Property	CP	CP	CBP	CBP	State I	State I	State II	State II	State III	State III
Pt–N1 (CBP)	–	–	2.110	2.081	2.089	2.076	2.103	2.074	2.096	2.082
Pt–N2 (CBP)	–	–	2.109	2.079	2.087	2.075	2.102	2.075	2.085	2.071
Pt–N3 (CP)	2.109	2.079	–	–	2.093	2.078	2.098	2.077	2.089	2.077
Pt–N 4(CP)	2.108	2.079	–	–	2.092	2.080	2.101	2.076	2.093	2.071
Pt–Cl1	2.343	2.379	–	–	2.375	2.393	2.355	2.387	2.349	2.384
Pt–Cl2	2.342	2.379	–	–	2.375	2.394	2.355	2.389	2.367	2.383
Pt–O1	–	–	2.015	2.046	2.047	2.064	2.024	2.056	2.023	2.048
Pt–O2	–	–	2.014	2.046	2.048	2.061	2.026	2.052	2.026	2.050
N1–H1	–	–	1.027	1.028	1.046	1.376	1.027	1.028	1.032	1.028
N2–H2	–	–	1.027	1.028	1.046	1.038	1.027	1.028	1.028	1.028
N3–H3	1.027	1.028	–	–	1.044	1.040	1.038	1.044	1.049	1.046
N4–H4	1.027	1.028	–	–	1.046	1.040	1.038	1.044	1.038	1.040
C–O1	–	–	1.343	1.323	1.345	1.330	1.335	1.316	1.329	1.321
C–O2	–	–	1.344	1.324	1.346	1.330	1.335	1.316	1.339	1.323
C=O3	–	–	1.232	1.247	1.233	1.244	1.238	1.253	1.250	1.252
C=O4	–	–	1.232	1.247	1.233	1.244	1.239	1.253	1.232	1.246
E_ad_ (Kcal/mol)	–	–	–	–	-33.82	− 14.69	− 13.65	− 12.47	− 28.38	− 14.27
E_sol_ (Kcal/mol)	–	–	–	–	–	19.13	–	1.18	–	14.11
ΔH (Kcal/mol)	–	–	–	–	− 33.89	− 8.23	− 13.31	− 5.88	− 28.26	− 7.80
ΔG (Kcal/mol)	–	–	–	–	− 20.04	4.67	− 1.53	5.17	− 15.49	4.52
ΔS (cal mol^-1^ k^−1^)	–	–	–	–	− 46.45	− 40.00	− 39.00	− 40.00	− 43.00	− 40.00
DM (Debye)	10.69	16.13	13.12	20.80	4.20	6.20	26.60	37.89	4.84	9.38
E_HOMO_	− 5.30	− 5.6	− 5.32	− 5.5	− 5.04	− 4.76	− 4.32	− 5.47	− 5.44	− 5.58
E_LUMO_	− 2.72	− 2.74	− 2.36	− 2.15	− 2.88	− 2.73	− 3.19	− 2.57	− 2.63	− 2.66
E_g_	2.58	2.86	2.96	3.35	2.16	2.03	1.13	2.90	2.81	2.92
ΔE_g_(%)	–	–	–	–	16.28	29.02	56.20	1.40	8.91	2.10
μ	− 4.01	− 4.17	− 3.84	− 3.83	− 3.96	− 3.75	− 3.76	− 4.02	− 4.04	− 4.12
η	1.29	1.43	1.48	1.68	1.08	1.02	0.57	1.45	1.41	1.46
χ	4.01	4.17	3.84	3.83	3.96	3.75	3.76	4.02	4.04	4.12
S	0.39	0.35	0.34	0.30	0.46	0.49	0.88	0.34	0.36	0.34
*ω*	6.23	6.08	4.98	4.37	7.26	6.91	12.48	5.57	5.79	5.81

The MEP analysis of CP and CBP, as depicted in Fig. 1, reveals distinct regions of electrophilic and nucleophilic character within these two drugs, providing a foundation for their potential intermolecular interactions. The MEP maps indicate that CP exhibits high electron density (red regions) around its chloride ions, highlighting their electronegative nature and propensity for participating in electrostatic interactions. In contrast, the hydrogen atoms attached to nitrogen ligands in both CP and CBP appear in blue regions, signifying low electron density and a more electrophilic character. For CBP, the oxygen atoms in the carbonyl groups also display high electron density (red regions), indicating their capacity to engage in nucleophilic interactions with electrophilic centers. These complementary electronic properties create opportunities for intermolecular interactions between CP and CBP. For instance, the electrophilic hydrogen atoms in CP could potentially form weak hydrogen bonds with the nucleophilic oxygen atoms of the carbonyl groups in CBP. Additionally, the electron-rich chloride ions in CP might interact with the relatively electron-poor hydrogen atoms in CBP, facilitating the formation of stable [CP- CBP] complexes.

**Fig. 2:**
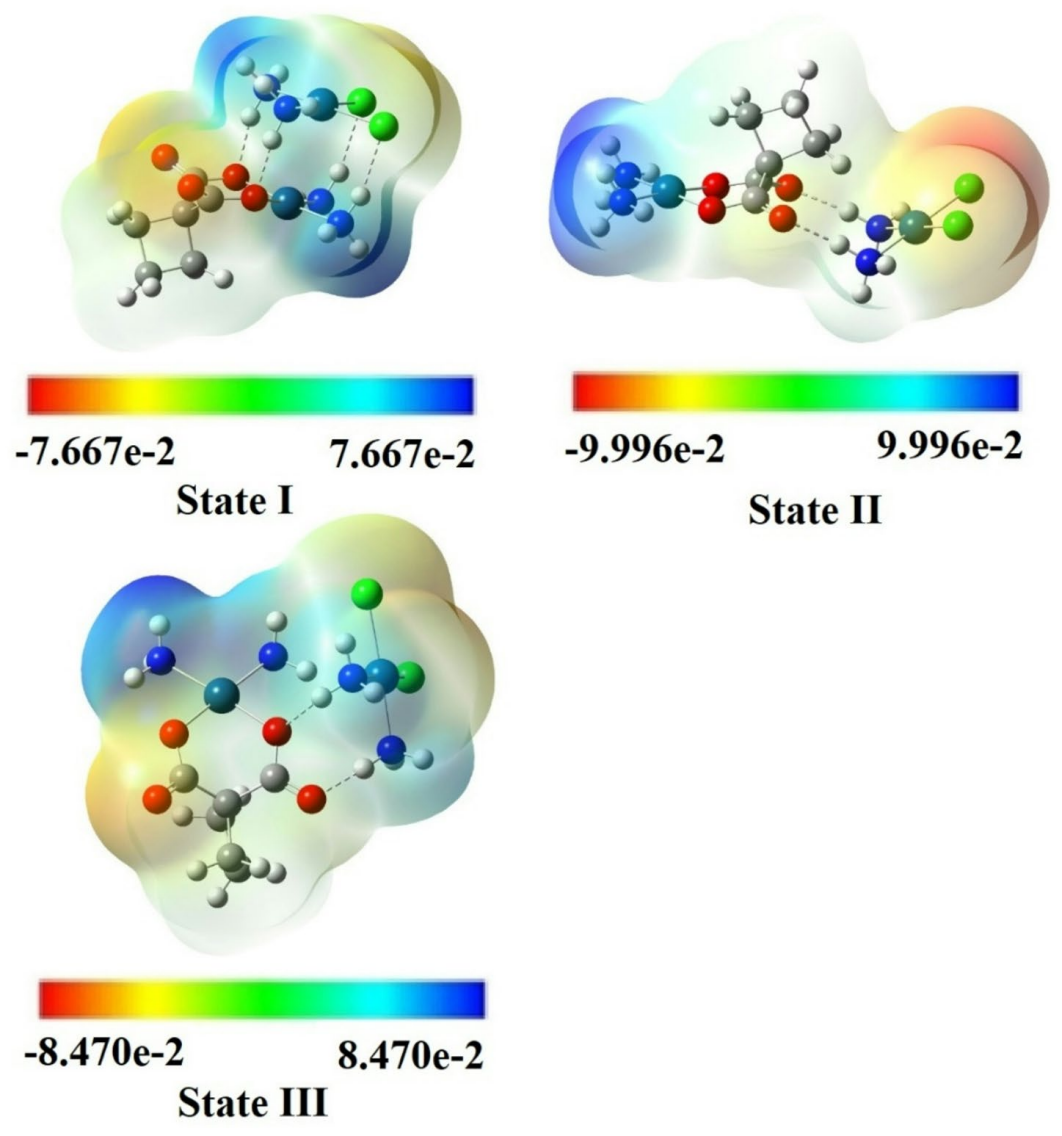
The MEP plots of the different configurations of the [CP–CBP] complexes.

Based on the various intermolecular interactions between the two drugs, different forms of [CP–CBP] complexes were investigated with respect to their biological activities. The MEP of three stable configurations of the [CP–CBP] complex, derived from hydrogen bonding, electrostatic interactions, and potential coordination, are depicted in Fig. 2. The MEP analysis further supports these configurations by highlighting the electron-rich and electron-poor regions, which may facilitate specific interactions with biological targets. The favorable electron density distribution in these stable complexes suggests potential for enhanced biological activity, as the interactions between the drug components may influence their affinity for cellular targets or biomolecules, potentially improving the efficacy of these complexes in cancer treatment.

**Fig. 3:**
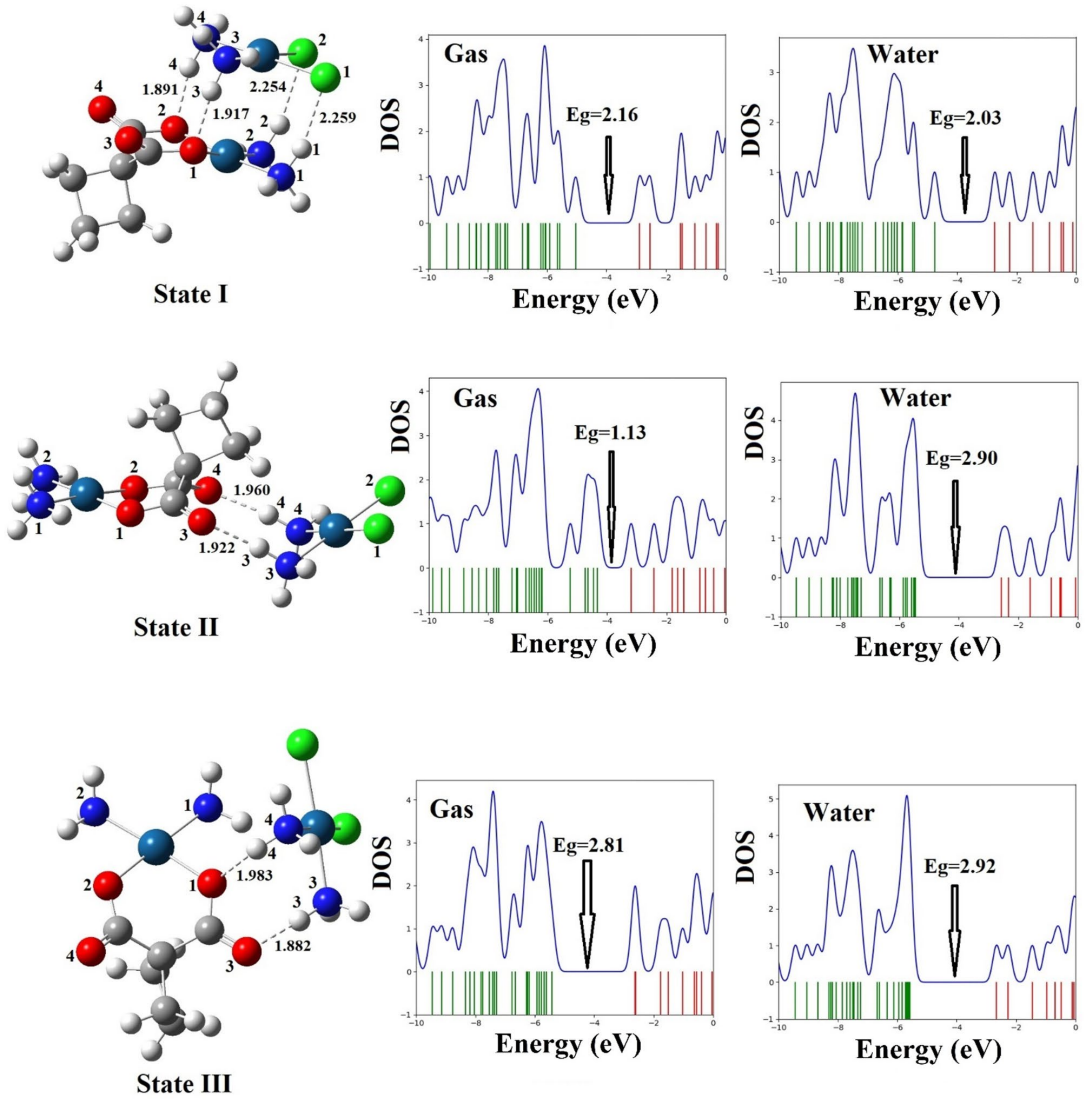
Structures optimized with equilibrium distances and DOS spectra of the [CP–CBP] complexes in the gas and water phases.

Based on the MEP results, three CP–CBP complexes were formed, as illustrated in Table 2 and Fig. 3 of this study, the interaction between CP and CBP was analyzed through three distinct configurations (State I, II, III). These configurations depict potential interaction modes between the two platinum-based drugs, and their structural, thermodynamic, and electronic properties were thoroughly investigated. The results suggest that the formation of CP–CBP complexes significantly alters their physicochemical characteristics, which may contribute to their enhanced therapeutic potential. The geometric optimization of the CP–CBP complexes revealed only slight variations in bond lengths compared to the isolated drugs (Table 2), indicating minimal structural distortion upon complex formation. Specifically, the Pt–N and Pt–Cl distances in CP, as well as the Pt–N and Pt-O distances in CBP, exhibited minor adjustments. For CP, the Pt–N bond lengths showed slight variations from 2.108 and 2.109 Å (gas) and 2.079 Å (aqueous) in the free drug to a range of 2.092–2.101 Å (gas) and 2.071–2.080 Å (aqueous) across the complexes. Similarly, the Pt–Cl bond lengths increased slightly from 2.342 and 2.343 Å (gas) and 2.379 Å (aqueous) to 2.349–2.375 Å (gas) and 2.383–2.394 Å (aqueous). For CBP, the Pt–N bond lengths varied minimally from 2.109 and 2.110 Å (gas) and 2.079 and 2.081 Å (aqueous) in the free state to 2.085–2.103 Å (gas) and 2.071–2.082 Å (aqueous) in the complexes. The Pt-O bond length followed a similar trend, reflecting only minor structural adjustments. These minor modifications suggest that the formation of CP–CBP complexes induces subtle yet potentially meaningful alterations in molecular geometry. While the bond length variations are minimal, they may still contribute to enhanced intermolecular interactions, such as hydrogen bonding and electrostatic attractions, which could influence the stability and bioavailability of the complexes. Also, these bond length modifications indicate potential synergistic effects that may influence the pharmacokinetic and pharmacodynamic behaviors of the drugs^2^.

Based on the thermodynamic analysis presented in Table 2, the thermodynamic analysis reveals that all three complexes exhibit favorable stability, as evidenced by their negative ΔH values. Specifically, State I demonstrates ΔH values of − 33.89 kcal/mol in the gas phase and − 8.23 kcal/mol in the aqueous phase, while State III shows ΔH values of − 28.26 kcal/mol in the gas phase and − 7.80 kcal/mol in the aqueous phase. Similarly, State II exhibits ΔH values of − 13.31 kcal/mol in the gas phase and − 5.88 kcal/mol in the aqueous phase. These results indicate that all three complexes are thermodynamically stable, with State I showing the highest exothermicity and thus the greatest stability among the configurations. However, the positive ΔG values in the aqueous phase for all states (4.67 kcal/mol for State I, 5.17 kcal/mol for State II, and 4.52 kcal/mol for State III) suggest that additional factors such as cellular environment or binding interactions may influence their efficacy under physiological conditions. Overall, these findings confirm that all three complexes are thermodynamically stable, with varying degrees of stability depending on the specific configuration.

The adsorption energy analysis of CP–CBP complexes reveals that all three states (State I, II, III) exhibit favorable interactions, as indicated by their negative Ead values. In the gas phase, State I shows the highest adsorption energy (− 33.82 kcal/mol), followed by State III (− 28.38 kcal/mol) and State II (− 13.65 kcal/mol). In the aqueous phase, adsorption energies decrease for all states (State I: − 14.69 kcal/mol, State II: − 12.47 kcal/mol, State III: − 14.27 kcal/mol), attributed to solvation effects where water molecules compete with drug-drug interactions. From a drug delivery perspective, the reduced adsorption energy in the aqueous phase is advantageous, as it allows for controlled dissociation at the target site while maintaining stability during circulation. State II, despite having lower adsorption energy, demonstrates favorable thermodynamic properties (ΔG = − 1.53 kcal/mol in the gas phase) and an increased dipole moment (26.60 Debye in gas, 37.89 Debye in aqueous phase), suggesting improved solubility and bioavailability. These attributes make State II particularly suitable for controlled release and reduced systemic toxicity. Notably, carboplatin (CBP) acts as an effective carrier-like for cisplatin (CP), enhancing its stability and preventing premature degradation in physiological environments. This carrier-like role improves solubility, controls drug release, and reduces systemic toxicity, thereby optimizing therapeutic outcomes. The findings indicate that CP–CBP complexes, especially State II, present a promising strategy for targeted drug delivery in breast and cervical cancer treatment, leveraging synergistic interactions and optimized pharmacokinetics. Further experimental validation is needed to explore their clinical implications. Solvation energy (Esol) values showed a reduction compared to the free drugs, implying decreased aqueous solubility. For instance, Esol for State II was 1.18 kcal/mol, lower than the values for CP and CBP alone, suggesting increased hydrophobic interactions within the complexes. While reduced solubility could impact bioavailability, the moderate adsorption energies and favorable thermodynamic parameters suggest that the complexes remain viable for therapeutic applications.

Density of states (DOS) analysis, presented in Fig. 3, revealed significant changes in the electronic structure of CP–CBP complexes relative to the individual drugs. The HOMO–LUMO energy gap (Eg) narrowed upon complex formation, indicating increased chemical reactivity. For instance, State II exhibited an Eg of 1.13 eV (gas phase) and 2.90 eV (aqueous phase), corresponding to a percentage reduction (ΔEg%) of 56.20% in the gas phase and 1.40% in aqueous conditions compared to CP alone. This suggests enhanced electron transfer potential, which may facilitate stronger interactions with biological targets, thereby improving anticancer activity^5^. Quantum molecular descriptors further reinforce these findings. State II displayed a chemical potential (μ) of − 3.76 eV and an electrophilicity index (ω) of 12.48 in the gas phase, both of which are higher than those of CP (μ = − 4.01 eV, ω = 6.23) and CBP (μ = − 3.84 eV, ω = 4.98). These values suggest that the CP–CBP complexes have an increased tendency to interact with electron-rich biological targets, potentially enhancing DNA binding and cytotoxic activity^3^. Recent studies have similarly computed and discussed quantum reactivity descriptors and HOMO–LUMO energy gaps in related systems^48–50^, which provide a useful benchmark for comparison. Overall, the optimized CP–CBP configurations (States I, II, and III), demonstrate structural and electronic properties that could enhance their efficacy as chemotherapeutic agents. The combination of CP with CBP in complex form appears to produce synergistic effects, leading to improved stability, enhanced reactivity, and stronger interactions with cancer cell targets. These findings lay the groundwork for future experimental investigations into the therapeutic potential of CP–CBP complexes in breast and cervical cancer treatment. Furthermore, the chemical reactivity of the [CP–CBP] complexes was explicitly evaluated using the HOMO–LUMO energy gap (Eg). A smaller Eg value indicates higher chemical reactivity and lower kinetic stability, suggesting that complexation enhances electron transfer capability and potential biological interactions. This behavior supports the improved synergistic effect observed in the [CP–CBP] systems. In summary, the structural and thermodynamic analyses in this section show that [CP–CBP] complexation induces only minor changes in Pt–ligand bond lengths but markedly stabilizes the systems (negative ΔH and Ead) and enhances their electronic reactivity, especially in State II.

### Quantum chemical analysis of CP–CBP complexes: insights from QTAIM, ELF, and LOL

The QTAIM, ELF, and LOL analyses provide valuable insights into the electronic structure, bonding characteristics, and potential biological relevance of the CP–CBP complexes. These computational approaches elucidate the nature of hydrogen bonding, charge delocalization, and intermolecular interactions, which play a key role in the stability, reactivity, and synergistic effects of the complexes. The presence of well-defined bond critical points (BCPs) in the QTAIM analysis, as presented in Table 3, confirms the formation of significant non-covalent interactions between CP and CBP, which contribute to the enhanced stability and potential therapeutic efficacy of these complexes.

**Table 3:** Topological parameters of hydrogen bonding and electronic interactions in CP–CBP complexes based on QTAIM, ELF, and LOL analyses in the water phase and at the bond critical points (BCPs).

Complex	Bond	H(r)	∇^2^ρ	ρ	G(r)	|V(r)|	Gr/|V|(r)	ELF	LOL
State I	H1….Cl1	0.00073	0.04424	0.01615	0.01032	0.00959	1.07655	0.07589	0.22291
	H2….Cl2	0.00062	0.04558	0.01694	0.01078	0.01016	1.06096	0.08128	0.22941
	H3…..O1	− 0.00056	0.07013	0.02445	0.01809	0.01865	0.96997	0.09641	0.24633
	H4…..O2	− 0.00058	0.07266	0.02533	0.01875	0.01933	0.96999	0.10058	0.25070
State II	H3…..O3	− 0.00002	0.08761	0.02924	0.02193	0.02195	0.99892	0.11658	0.26656
	H4…..O4	0.00000	0.08991	0.02992	0.02247	0.02247	1.00011	0.11938	0.26920
State III	H4…..O1	− 0.00042	0.08361	0.02830	0.02133	0.02174	0.98083	0.11121	0.26139
	H3…..O3	− 0.00074	0.08232	0.02991	0.02133	0.02207	0.96621	0.13078	0.27958

Data values are given in atomic units (a.u.).

Among the three configurations, State II emerges as the most stable, exhibiting higher electron density (ρ) at hydrogen bond sites, such as H3⋯O3 (0.02924 a.u.) and H4⋯O4 (0.02992 a.u.), suggesting stronger intermolecular interactions and enhanced complex stability. However, State I and State III also contribute to the overall synergistic effect. In State I, the presence of H1⋯Cl1 and H2⋯Cl2 interactions, with moderate electron density (ρ) and Laplacian values (∇^2^ρ), indicates electrostatic stabilization, which could play a role in drug solubility and systemic circulation. State III, with H3⋯O3 and H4⋯O1 interactions, exhibits high ELF and LOL values, suggesting enhanced charge delocalization and improved bio molecular interaction potential.

**Fig. 4:**
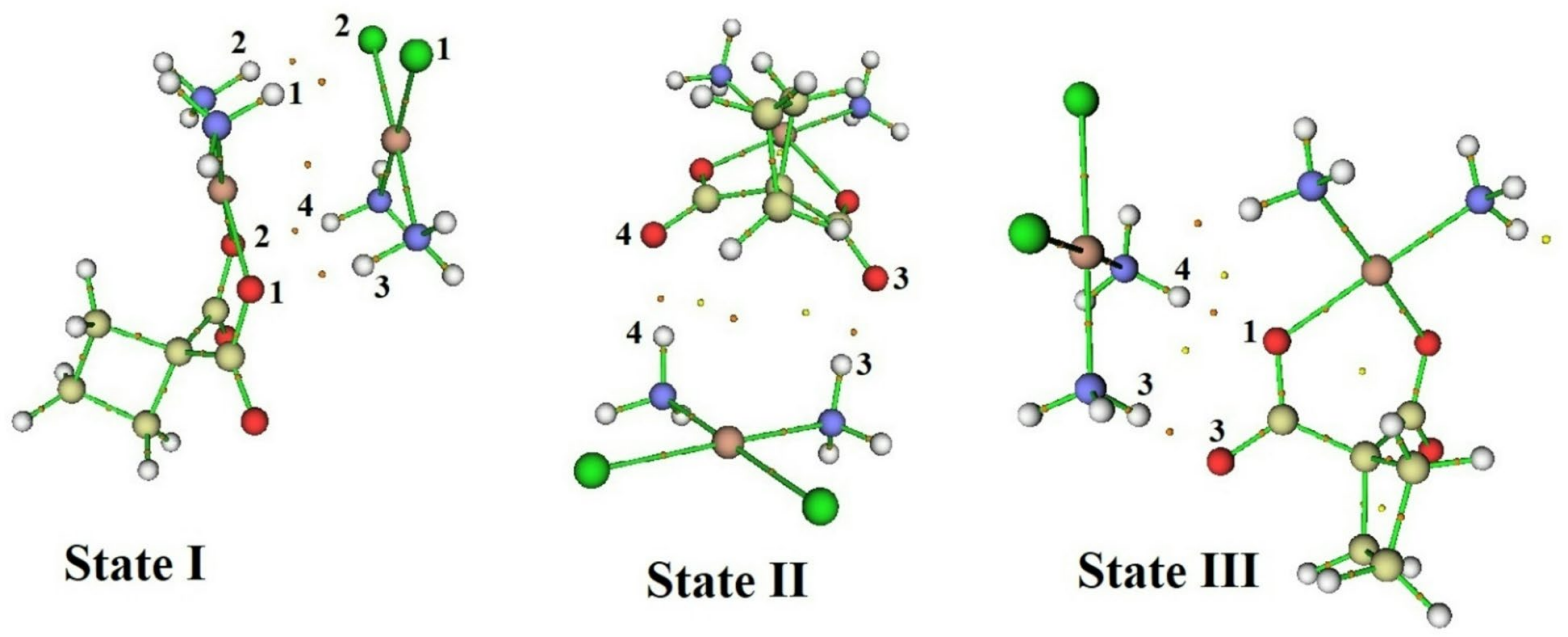
QTAIM molecular graphs depicting bond critical points and electron density distribution in CP–CBP complexes in the water phase.

The Laplacian of electron density (∇^2^ρ) values in Table 3 further confirm the closed-shell nature of these interactions, particularly in State II and State III, where positive Laplacian values indicate dominant hydrogen bonding. Additionally, the total energy density (H(r)) values in State II are close to zero, reflecting a balance between kinetic and potential energy contributions, which supports greater charge delocalization and increased reactivity. The Gr/|V|(r) ratio, which provides insight into bond strength, is closest to unity in State II, confirming that the hydrogen bonds in this configuration exhibit partial covalent character, further stabilizing the CP–CBP interaction network^51^. The QTAIM molecular graphs in Fig. 4 visually confirm these findings, highlighting the electron density distribution and the key interaction pathways that reinforce the formation of CP–CBP complex.

**Fig. 5:**
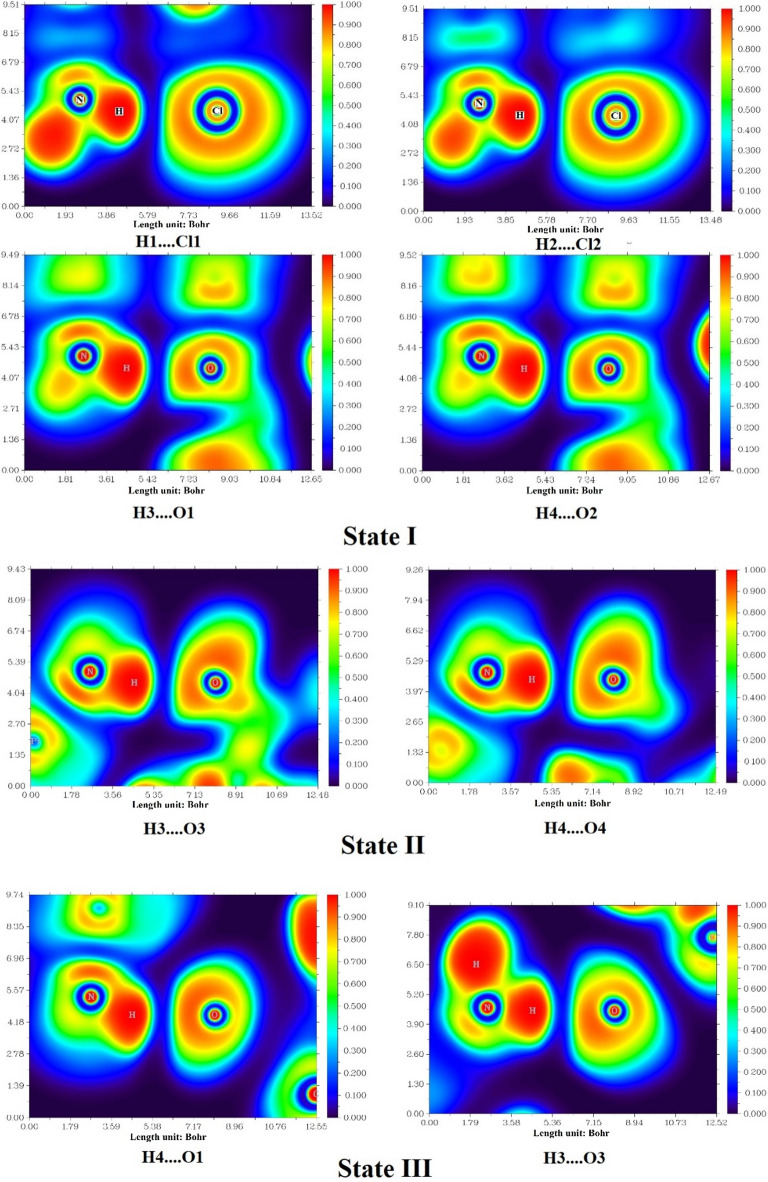
ELF surface maps illustrating electron localization in different configurations of CP–CBP complexes in the water phase.

**Fig. 6:**
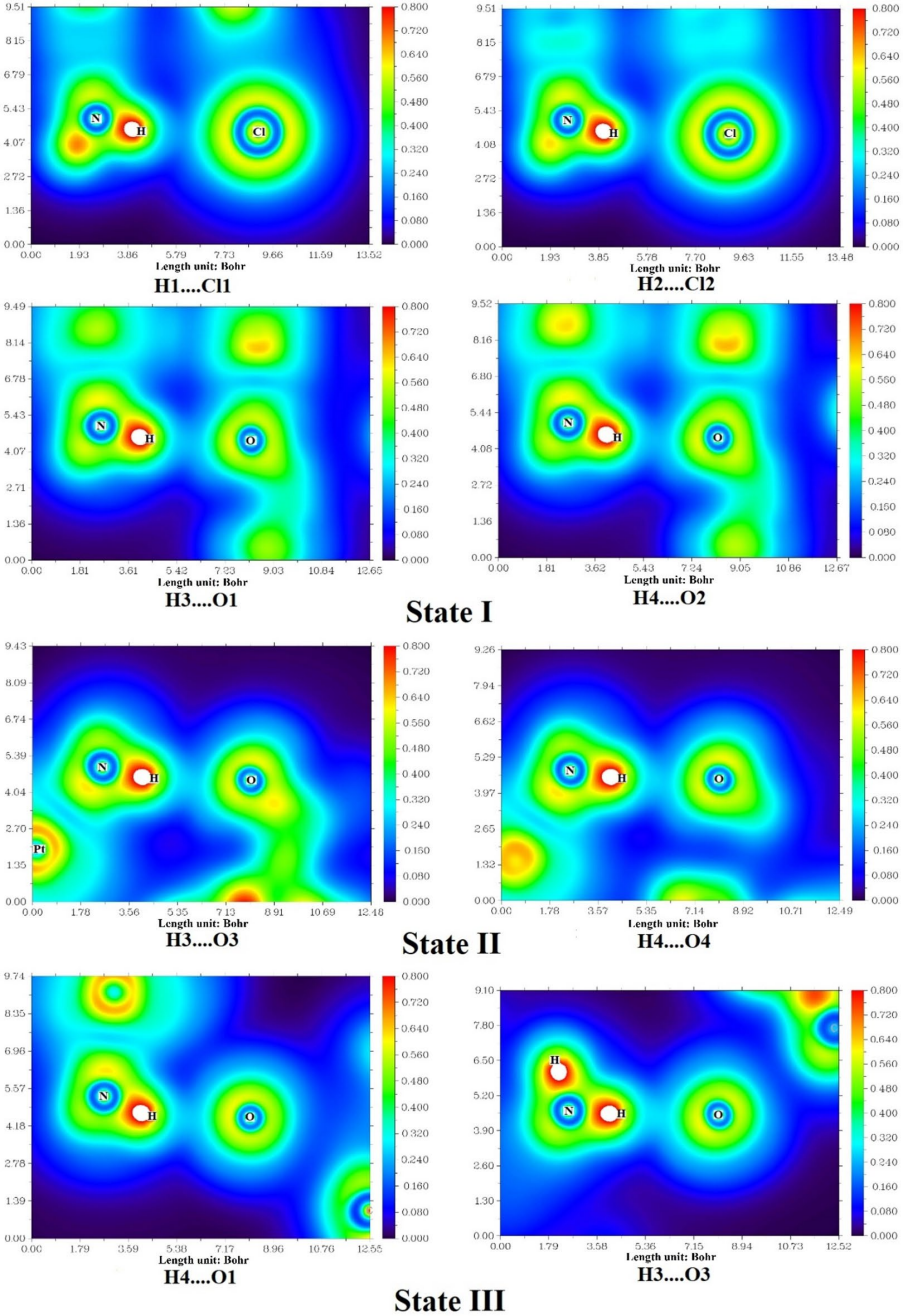
LOL surface maps highlighting orbital interactions and charge delocalization in CP–CBP complexes in the water phase.

The ELF and LOL analyses, as illustrated in Figs. 5 and 6, provide significant insights into the bonding nature and electron delocalization within the CP–CBP complexes in states I, II, and III. These computational descriptors highlight regions of high electron localization, aiding in identifying key interaction sites^52^. The ELF values, ranging between 0 and 1, and LOL values, spanning up to 0.800, delineate the extent of charge localization and orbital interactions. Notably, in the ELF surface maps, State II exhibits higher ELF values (0.11658–0.11938 a.u.), indicating enhanced charge stability, whereas State III displays the most pronounced LOL values (0.26656–0.27958 a.u.), reflecting stronger orbital overlap. The predominance of electron delocalization below 0.5 in all states aligns well with QTAIM analysis, which confirms that hydrogen bonding and van der Waals forces govern the CP–CBP interactions. These extensive delocalization effects could facilitate a smoother drug release profile at target sites, potentially optimizing therapeutic efficacy. Collectively, ELF and LOL analyses reinforce the potential synergistic effect of CP–CBP complexes in chemotherapy by elucidating their electronic and bonding behaviors.

The modifications in structural and electronic properties align well with key physicochemical parameters, as presented in Table 2. The Pt–N and Pt–O bond length variations suggest that the interaction between CP and CBP modulates coordination geometry, leading to greater stability and altered pharmacokinetic behavior. Furthermore, the HOMO–LUMO gap (Eg) reduction in State II (1.13 eV in gas phase, 2.90 eV in aqueous phase) and State III (2.81 eV in gas phase, 2.92 eV in aqueous phase) suggests increased reactivity and stronger biomolecular interactions, which could enhance anticancer efficacy^3^. These findings collectively indicate that the synergistic effect of CP and CBP is the result of contributions from all three configurations. While State II exhibits the strongest hydrogen bonding network and electronic stability, State I enhances solubility and bioavailability, and State III contributes to charge delocalization and biomolecular interactions. This suggests that the co-administration of CP and CBP in complex form may optimize therapeutic outcomes, enhancing drug retention, cellular uptake, and DNA-binding efficiency^6^. Overall, these computational insights suggest that CP–CBP complexes could serve as a promising chemotherapeutic strategy, benefiting from stabilizing hydrogen bonds (State II), electrostatic interactions (State I), and strong charge delocalization (State III). The thermodynamic, electronic, and bonding analyses support the hypothesis that CP–CBP complexes may enhance anticancer activity, warranting further in vitro and in vivo validation to confirm their clinical potential. In summary, the QTAIM, ELF, and LOL analyses consistently show that (i) hydrogen-bonding networks, strongest in State II, are the dominant stabilizing interactions, (ii) electrostatic H···Cl contacts in State I and pronounced charge delocalization in State III complement this stabilization, and (iii) these cooperative noncovalent effects rationalize the enhanced stability and reactivity of the [CP–CBP] complexes compared with the isolated drugs.

### UV–vis analysis and frontier molecular orbitals

**Fig. 7:**
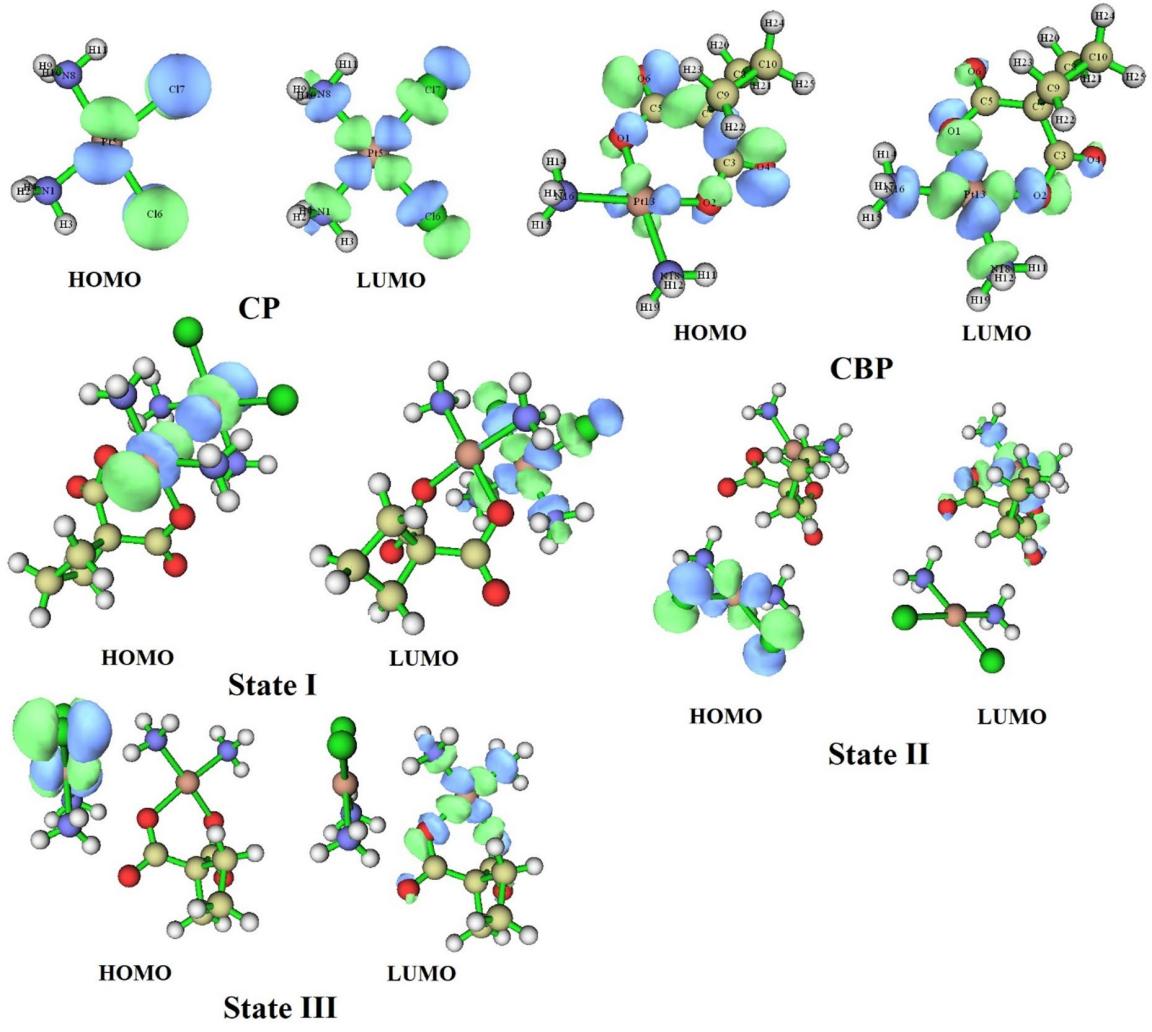
HOMO and LUMO energy level diagrams of CP, CBP, and their complexes (State I, II, III) in aqueous environments.

**Fig. 8:**
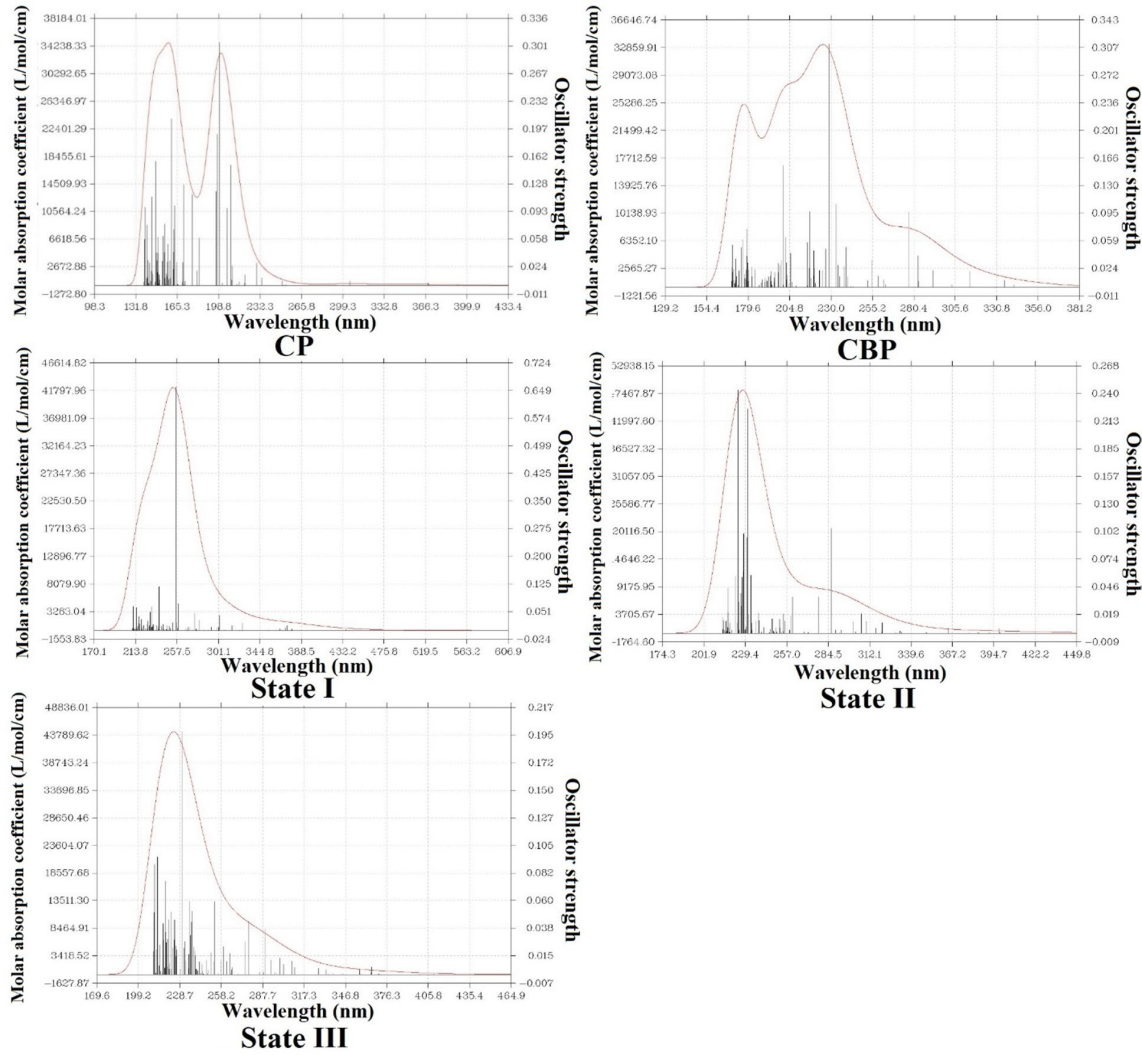
Theoretical UV–Vis spectra of CP, CBP, and their complexes (State I, II, III) in aqueous environments.

The UV–Vis absorption spectra and frontier molecular orbitals (HOMO–LUMO) of CP, CBP, and their CP–CBP complexes (States I, II, and III) were analyzed to investigate their electronic transitions and optical behavior. The excitation energies (E, eV), maximum absorption wavelengths (λmax, nm), and oscillator strengths (f) are summarized in Table 4, while the HOMO and LUMO distributions are depicted in Fig. 7, and the UV–Vis spectra in Fig. 8.

**Table 4:** Optoelectronic properties of CP, CBP, and their complexes (State I, II, III) in aqueous environments.

Compound	E/eV	λ_max_/nm	*f*
Cp	6.222	199.41	0.3056
	6.274	197.74	0.1901
	5.984	208.58	0.1514
CBP	5.423	228.77	0.3114
	6.185	200.60	0.1560
State I	4.839	256.41	0.6581
State II	5.523	224.62	0.2438
	5.375	230.81	0.2250
State IIII	5.386	230.34	0.1973

The HOMO–LUMO gap (Eg) is a critical parameter in understanding molecular stability and reactivity. As illustrated in Fig. 7, CP and CBP exhibit Eg values of 2.86 eV and 3.35 eV in the aqueous phase, respectively, indicating their relative electronic stability. However, upon complexation, notable changes are observed. State I exhibits a significant Eg reduction to 2.03 eV in water, suggesting enhanced electronic delocalization and increased chemical reactivity. State II displays a moderate Eg drop to 2.90 eV in water, highlighting a more reactive and potentially biologically interactive complex. State III retains an Eg value of 2.92 eV in water, closer to CBP, indicating a balanced electronic redistribution without excessive destabilization. These trends align with UV–Vis absorption shifts, reinforcing that lower Eg values lead to redshifted optical transitions.

The UV–Vis spectra (Fig. 8) reveal that CP has a primary absorption peak at 199.41 nm (6.222 eV, f = 0.3056), while CBP absorbs at 228.77 nm (5.423 eV, f = 0.3114). Previous experimental studies on the UV–Vis spectra of CP and CBP as individual drugs have reported absorption maxima (λmax) values consistent with our computational findings. For example, CP typically absorbs around 200 nm, aligning well with our computed λmax values of 199.41 nm and 197.74 nm^3^. Similarly, CBP’s experimental λmax near 230 nm corroborates our calculated value of 228.77 nm. This agreement validates the accuracy of our TD-DFT calculations and highlights the reliability of the BP86 functional combined with the LANL2DZ/6–31 + G** basis set for predicting the optoelectronic properties of platinum-based drugs^16^. The observed redshift in CP–CBP complexes suggests increased conjugation and charge transfer interactions. State I exhibits a pronounced redshift in water, with λmax at 256.41 nm (4.839 eV, f = 0.6581), correlating with its substantial Eg reduction. This suggests a higher degree of electronic communication between CP and CBP, favoring charge transfer interactions. State II displays moderate blueshifts with two main absorption peaks at 224.62 nm (5.523 eV, f = 0.2438) and 230.81 nm (5.375 eV, f = 0.2250), consistent with its intermediate Eg value. This reflects a more controlled electronic delocalization while maintaining molecular integrity. State III retains absorption in a similar range to CBP, with λmax = 230.34 nm (5.386 eV, f = 0.1973), indicating a stable electronic configuration with limited perturbation from complexation.

The correlation between HOMO–LUMO gap narrowing (Fig. 7) and UV–Vis spectral shifts (Fig. 8) suggests that complexation enhances electronic interactions, potentially improving biological activity. State I, with the largest Eg reduction (2.03 eV in water) and strongest absorption at 256.41 nm, exhibits the highest electronic communication, which may facilitate stronger DNA binding and anticancer efficacy. State II, with a more balanced Eg and UV–Vis response, could offer an optimal trade-off between reactivity and stability, making it a promising candidate for enhanced therapeutic application. State III, with minimal spectral shifts, suggests that its electronic structure remains close to that of CBP, indicating that this state retains characteristics of the individual drug molecules. In conclusion, the UV–Vis analysis confirms that the formation of [CP–CBP] complexes significantly modifies the optoelectronic properties of the individual drugs. These changes could enhance their therapeutic efficacy in treating breast and cervical cancers by promoting stronger interactions with biomolecular targets. Future experimental studies are warranted to validate these computational predictions and explore the clinical potential of these complexes.

### Infrared spectroscopy analysis of molecular interactions and structural changes in CP–CBP complexes

**Fig. 9:**
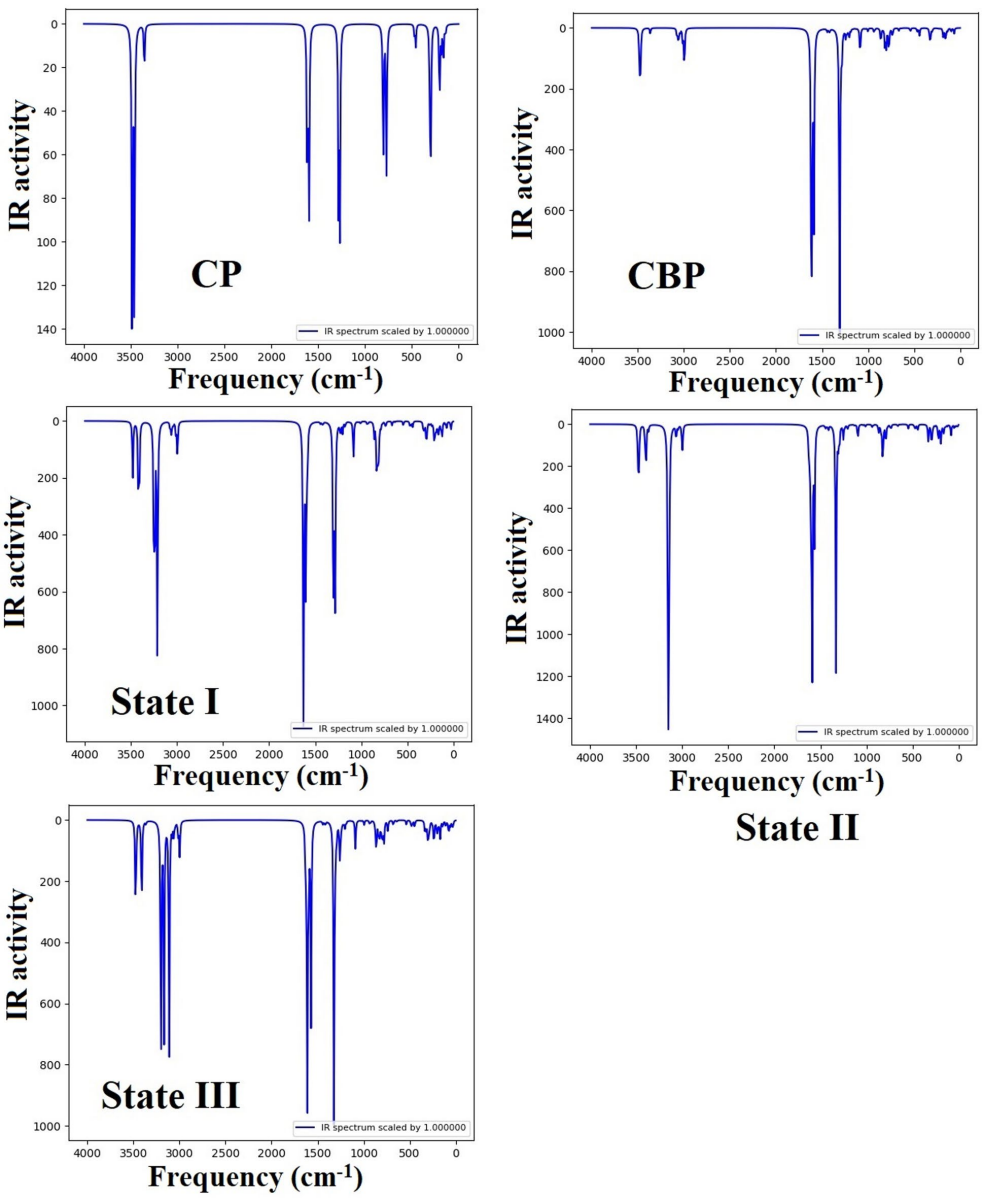
The IR curves of cisplatin (CP) and carboplatin (CBP) drugs and as well as State I, II, and III in the [CP–CBP] complexes in the water environments.

Infrared (IR) spectroscopy plays a crucial role in characterizing the molecular structure and interactions within drug complexes. The vibrational spectra of CP, CBP, and their CP–CBP complexes (States I, II, and III) reveal significant changes in bonding characteristics, indicating strong intermolecular interactions. The observed shifts in key vibrational modes, as summarized in Table 5, suggest structural modifications that enhance the stability of the CP–CBP complexes. The complete IR spectra of CP, CBP, and their complexes are depicted in Fig. 9, which illustrates the frequency shifts due to complex formation.

**Table 5:** Key infrared vibrational modes and observed shifts in CP, CBP, and CP–CBP complexes in water phase.

Vibrational mode	CP (cm⁻^1^)	CBP (cm⁻^1^)	State I (cm⁻^1^)	State II (cm⁻^1^)	State III (cm⁻^1^)	Observed shift	Interpretation
ν(Pt–N)	472.66	465.64	475.31 (470.51)	484.22 (475.17)	489.48 (476.11)	Blue shift	Strengthening of Pt–N bond due to ligand interaction or hydrogen bonding effects
ν(Pt–O)	–	540.28	542.75	546.30	536.47	Red shift (State I and II) Blue shift (state III)	Coordination effects in CP–CBP complexes
ν(C=O)	–	1587.31	1601.80	1563.97	1570.85	Blue shift in State I and Red shift in State II and III	Hydrogen bonding weakens the C=O bond
ν(N–H)	3355.51	3364.34	3209.28 (3240.74)	3140.94 (3365.57)	3111.81 (3363.95)	Red shift	Depending on hydrogen bonding strength
ν(Pt–Cl)	306.53	–	294.36	295.76	302.12	Red shift	complexation effects

The values in parentheses represent the vibrational frequencies of CBP for comparison with CP in the corresponding states.

The Pt–N stretching mode, a key marker of platinum coordination, appears at 472.66 cm⁻^1^ in CP and 465.64 cm⁻^1^ in CBP. Upon complexation, this vibration undergoes a blue shift, increasing to 475.31 cm⁻^1^ in State I, 484.22 cm⁻^1^ in State II, and 489.48 cm⁻^1^ in State III. This shift suggests an enhancement in Pt–N bond strength, likely due to increased ligand interaction and hydrogen bonding effects. A similar trend is observed in the Pt–O stretching mode of CBP, which shifts from 540.28 to 546.30 cm⁻^1^ in State II, indicating stronger hydrogen bonding interactions. Interestingly, State III shows a slight red shift (536.47 cm⁻^1^), suggesting a different interaction mode, possibly due to different interactions.

The C=O stretching frequency, a hallmark of carbonyl group interactions in CBP, exhibits a blue shift in State I (1601.80 cm⁻^1^) but a red shift in States II (1563.97 cm⁻^1^) and III (1570.85 cm⁻^1^). This suggests differential hydrogen bonding effects, where in State I, ligand interaction strengthens the C=O bond, whereas in States II and III, hydrogen bonding weakens it. These variations highlight the dynamic nature of the CP–CBP complexation process, where different configurations lead to distinct electronic and structural modifications.

A pronounced red shift is observed in the N–H stretching vibrations, which appear at 3355.51 cm⁻^1^ in CP and 3364.34 cm⁻^1^ in CBP. In the CP–CBP complexes, this frequency decreases significantly to 3209.28 cm⁻^1^ (State I), 3140.94 cm⁻^1^ (State II), and 3111.81 cm⁻^1^ (State III), indicating the involvement of N–H bonds in strong hydrogen bonding interactions. This effect is particularly evident in State III, which shows the lowest frequency shift, suggesting the strongest hydrogen bonding network.

Finally, the Pt–Cl stretching mode, a characteristic vibration of CP at 306.53 cm⁻^1^, shifts downward in all complex states. This red shift is most prominent in State I (294.36 cm⁻^1^) and State II (295.76 cm⁻^1^), indicating a weakening of Pt–Cl interactions due to coordination with CBP. Conversely, State III exhibits a milder shift (302.12 cm⁻^1^), suggesting a more balanced interaction that retains some characteristics of free CP. The overall vibrational spectral shifts observed in Fig. 9 confirm significant structural changes upon CP–CBP complexation. The strong hydrogen bonding and coordination effects, particularly in States II and III, suggest enhanced molecular stability, which could influence the pharmacokinetics and bioactivity of these drug complexes. These findings align with previous computational and experimental studies on platinum-based chemotherapeutics, reinforcing the potential of CP–CBP complexes in cancer treatment applications^3,16^.

### Molecular docking analysis of CP, CBP, and their complexes for breast and cervical cancer treatment

**Fig. 10:**
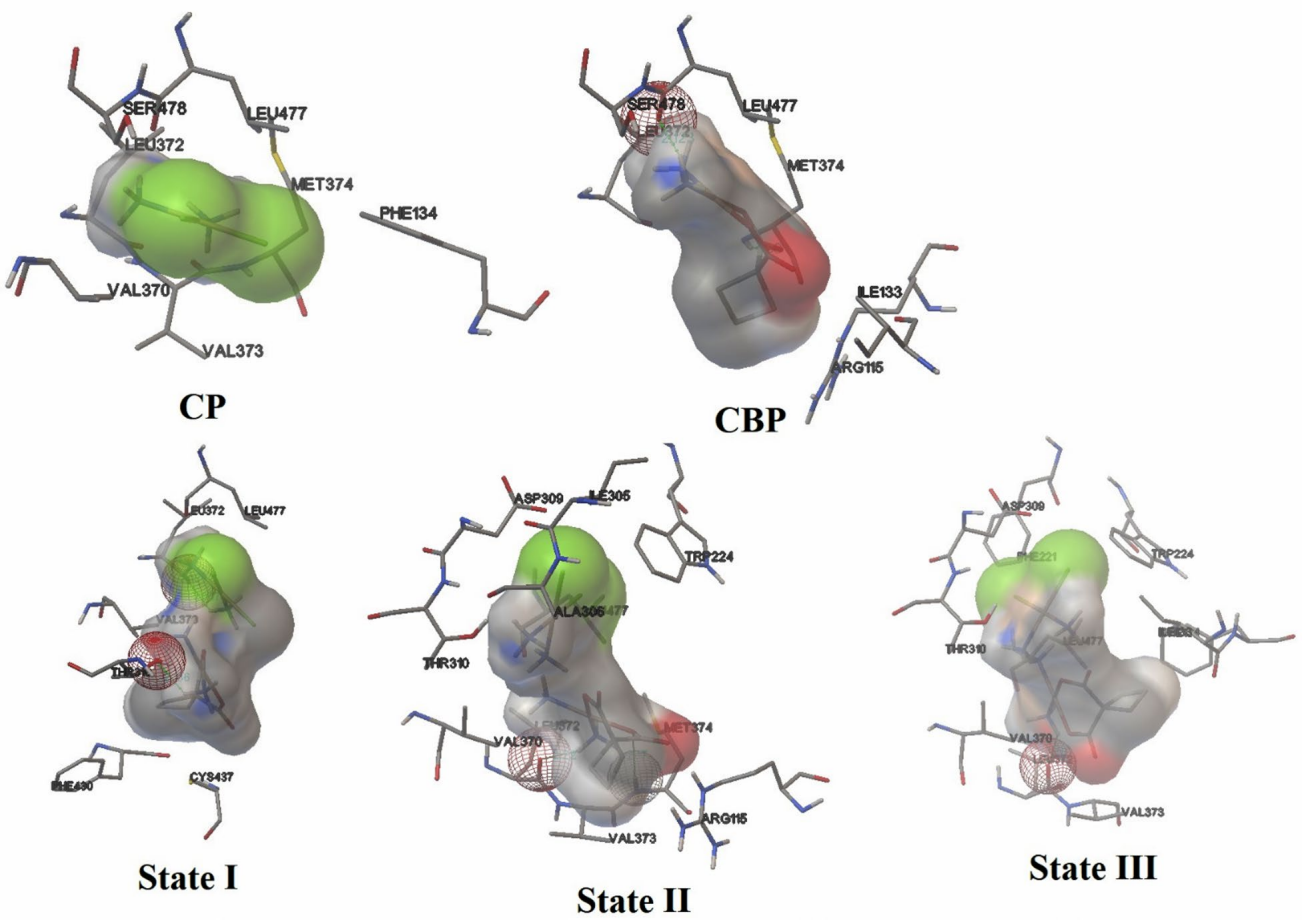
Visual representation of intermolecular hydrogen bonding interactions between CP, CBP, and their [CP–CBP] complexes within the active site of human placental aromatase cytochrome P450 19A1 (PDB ID: 3EQM).

**Fig. 11:**
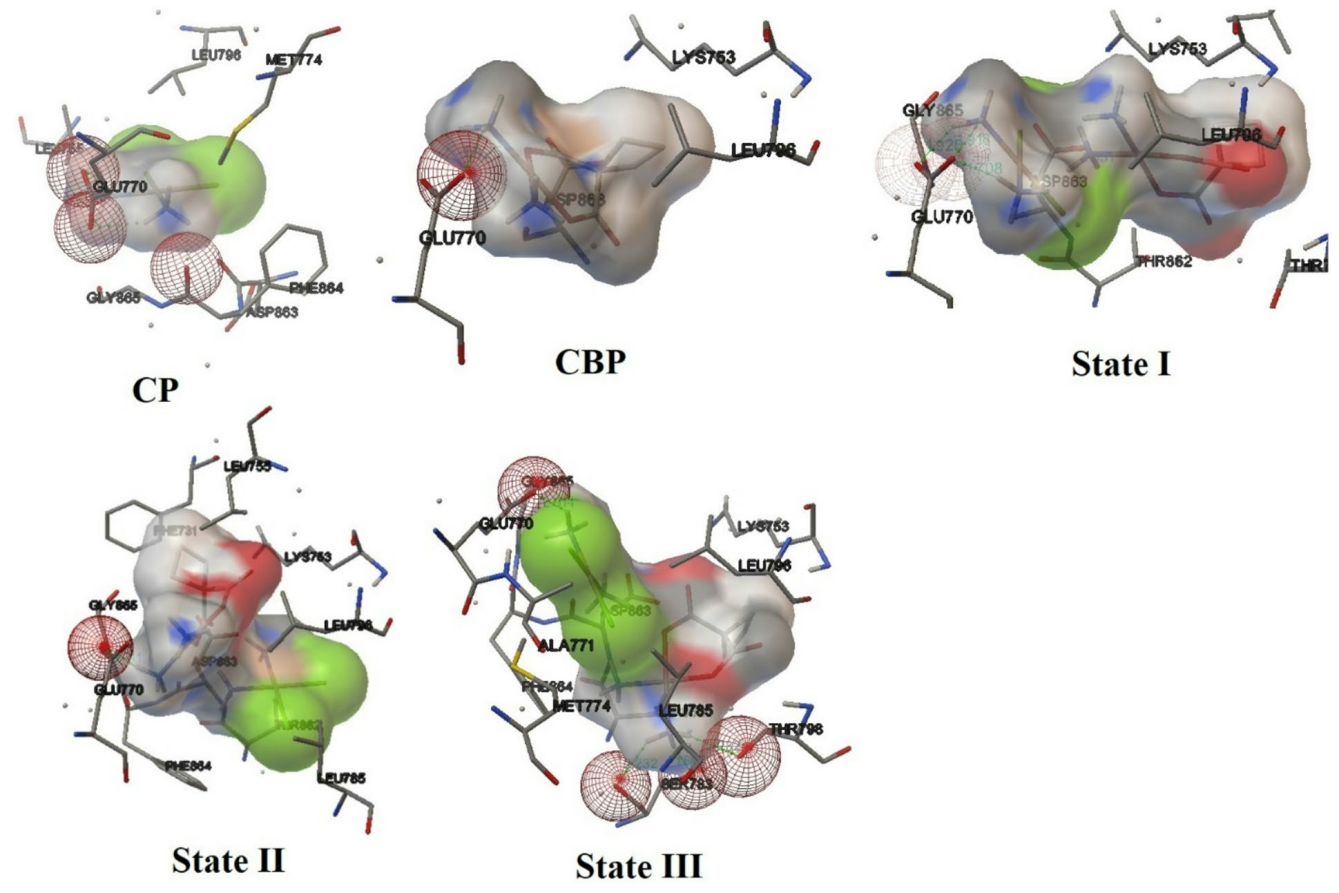
Visual representation of intermolecular hydrogen bonding interactions between CP, CBP, and their [CP–CBP] complexes within the active site of human epidermal growth factor receptor 2 (HER2) (PDB ID: 3RCD).

The molecular docking analysis was conducted to evaluate the binding affinities and interaction patterns of CP, CBP, and their complexes ([CP–CBP]) with key protein targets implicated in breast and cervical cancers: human placental aromatase cytochrome P450 19A1 (PDB ID: 3EQM) and human epidermal growth factor receptor 2 (HER2) (PDB ID: 3RCD). The results are summarized in Table 6, while Figs. 10 and 11 visually represent the hydrogen bonding interactions within the active sites. When comparing the individual drugs for aromatase (PDB ID: 3EQM), CP exhibited moderate binding affinity (E_Doc_ = − 2.57 kcal/mol) but lacked hydrogen bonds, interacting primarily with residues such as MET374, ARG115, PHE134, and VAL373. In contrast, CBP demonstrated stronger binding affinity (E_Doc_ = − 4.49 kcal/mol) and formed two hydrogen bonds with MET374 (1.890 Å) and LEU477 (2.123 Å). Despite its higher binding energy, CBP’s dissociation constant (Ki = 513.01 μM) indicates weaker binding strength compared to CP (Ki = 13.09 μM).

**Table 6:** Molecular docking analysis of CP, CBP, and their [CP–CBP] complexes at the active sites of human placental aromatase cytochrome P450 19A1 (PDB ID: 3EQM) and (HER2) (PDB ID: 3RCD).

Proteins	Compounds	*E*_Doc_ (Kcal/mol)	*K*_*i*_(uM)	NOH-bonds	Interacting residues (H-bonds) and distances/Å	Active sites
3EQM	CP	− 2.57	13.09	–	–	MET374, ARG115, PHE134, VAL373
	CBP	− 4.49	513.01	2	MET374 (1.890), LEU477 (2.123)	
	State I	− 2.91	7.37	3	LEU372 (2.004, 2.057), THR310 (2.186)	
	State II	− 4.15	911.67	2	MET374 (2.170), LEU372 (2.22)	
	State III	− 3.75	1.78	1	LEU372 (1.917)	
3RCD	CP	− 3.26	4.08	4	GLU770 (1.972, 1.946, 2.191), GLY865 (1.926)	THR862, ASP863, LYS753
	CBP	− 5.01	211.98	1	Glu770 (1.825)	
	State I	− 4.50	502.45	4	GLU770 (1.925, 1.918, 1.708), ASP863 (1.931)	
	State II	− 5.48	96.86	1	Glu770 (2.041)	
	State III	− 4.24	784.20	4	SER783 (2.232, 1.825), THR798 (2.232), GLU770 (2.014)

Among the [CP–CBP] complexes, State III displayed the best performance for aromatase inhibition, with a binding affinity of − 3.75 kcal/mol and an exceptionally low Ki value of 1.78 μM. This state formed one stable hydrogen bond with LEU372 (1.917 Å), suggesting enhanced therapeutic potential through complexation. While State I also showed improved binding affinity (− 2.91 kcal/mol, Ki = 7.37 μM) and formed three hydrogen bonds with LEU372 (2.004 Å, 2.057 Å) and THR310 (2.186 Å), its intermediate Ki value suggests slightly lower efficacy compared to State III. Conversely, State II exhibited weaker performance (E_Doc_ = − 4.15 kcal/mol, Ki = 911.67 μM), forming only two hydrogen bonds with MET374 (2.170 Å) and LEU372 (2.22 Å).

In the case of HER2 (PDB ID: 3RCD), CP demonstrated superior binding affinity (E_Doc_ = − 3.26 kcal/mol) and a low Ki value (4.08 μM), forming four hydrogen bonds with GLU770 (1.972 Å, 1.946 Å, 2.191 Å) and GLY865 (1.926 Å). On the other hand, CBP showed stronger binding affinity (E_Doc_ = − 5.01 kcal/mol) but a significantly higher Ki value (211.98 μM), forming just one hydrogen bond with GLU770 (1.825 Å). These findings indicate that CP is more favorable for HER2 inhibition due to its lower Ki value, despite CBP’s higher binding energy. Among the [CP–CBP] complexes, State II emerged as the most promising candidate for HER2 inhibition, exhibiting the highest binding affinity (E_Doc_ = − 5.48 kcal/mol) and a relatively low Ki value (96.86 μM). This state formed one hydrogen bond with GLU770 (2.041 Å), indicating strong and specific interactions. State I also performed well (E_Doc_ = − 4.50 kcal/mol, Ki = 502.45 μM), forming four hydrogen bonds with GLU770 (1.925 Å, 1.918 Å, 1.708 Å) and ASP863 (1.931 Å). However, its high Ki value suggests reduced practical efficacy compared to State II. Lastly, State III demonstrated moderate binding affinity (E_Doc_ = − 4.24 kcal/mol, Ki = 784.20 μM), forming four hydrogen bonds with SER783 (2.232 Å, 1.825 Å), THR798 (2.232 Å), and GLU770 (2.014 Å).

Based on molecular docking results, cisplatin (CP) demonstrates stronger binding efficiency than carboplatin (CBP) due to its lower Ki values for both aromatase (3EQM) and HER2 (3RCD). While CBP exhibits higher binding energy, its elevated Ki values limit its practical efficacy. The [CP–CBP] complexes, however, show significant synergistic effects by combining the complementary properties of CP and CBP. For aromatase (3EQM), State III exhibits the strongest binding affinity and lowest Ki value, enhancing therapeutic potential for cervical cancer. For HER2 (3RCD), State II displays the highest binding energy and a favorable Ki value, surpassing both CP and CBP individually, offering potential benefits for breast cancer treatment. Although the docking binding affinities of the [CP–CBP] complexes were in the moderate range (− 2.5 to − 5.5 kcal/mol), such values are typical for metal-based systems and are generally weaker than those obtained for many high-affinity small-molecule HER2 or aromatase inhibitors that are specifically optimized for very strong target binding. Accordingly, in this work the docking scores are interpreted in a relative manner, by directly comparing the [CP–CBP] complexes with the parent drugs (cisplatin and carboplatin), rather than against clinically optimized organic inhibitors. While cisplatin and carboplatin individually exhibit distinct and limited binding tendencies, their hybrid complex demonstrates enhanced stability and complementary interactions, reflecting a cooperative (synergistic) effect that strengthens overall target affinity. This synergism results from the stabilizing role of carboplatin and the higher reactivity of cisplatin, producing a balanced complex with improved pharmacological potential. Overall, the [CP–CBP] complexes improve molecular stability, prevent premature degradation, and enhance antitumor potential through strengthened binding affinities and optimized hydrogen bonding interactions. In summary, the docking results indicate that (i) cisplatin generally binds more efficiently than carboplatin to both HER2 and aromatase (lower Ki values), and (ii) [CP–CBP] complexation, particularly in States II and III, combines the favorable features of both parent drugs and leads to improved binding behavior compared with CP and CBP alone. In conclusion, [CP–CBP] complexes, particularly State III for 3EQM and State II for 3RCD, represent promising chemotherapeutic agents for breast and cervical cancers, warranting further experimental validation.

## Conclusion

This study highlights the significant therapeutic potential of cisplatin–carboplatin [CP–CBP] complexes for breast and cervical cancer treatment. Through integrated computational approaches, DFT, QTAIM, molecular docking, and spectroscopic analyses, the physicochemical properties, electronic behavior, and biological interactions of the complexes were comprehensively elucidated. Structural optimization and MEP mapping revealed enhanced intermolecular interactions and complementary charge distributions that stabilize the hybrid complexes, while thermodynamic analyses confirmed their exothermic and favorable formation. Quantum descriptors, including the HOMO–LUMO energy gap, electrophilicity index, and chemical potential, indicated increased reactivity and synergistic electron delocalization, consistent with QTAIM, ELF, and LOL results showing strong hydrogen bonding and charge redistribution. UV–Vis and IR spectral analyses further validated these structural and electronic modifications, demonstrating altered electronic transitions and vibrational shifts upon complexation. Molecular docking confirmed improved binding affinities of [CP–CBP] complexes toward HER2 and aromatase compared to the individual drugs, supporting their enhanced anticancer potential. Carboplatin acts as a stabilizing carrier-like for cisplatin, improving solubility, bioavailability, and controlled release while mitigating toxicity. Collectively, these findings suggest that [CP–CBP] complexes represent a promising platinum-based chemotherapeutic strategy combining efficacy, stability, and reduced side effects. As this study is purely theoretical, its predictions are limited by the inherent approximations of DFT and docking models, which cannot fully capture biological complexity. Therefore, future experimental validation, including synthesis, spectroscopic characterization, and in vitro/in vivo testing, is essential to confirm the therapeutic feasibility of the [CP–CBP] complexes under physiological conditions.

## Data Availability

All data generated or analyzed during this study are included in this published article.
